# Tomato Diseases and Pests Detection Based on Improved Yolo V3 Convolutional Neural Network

**DOI:** 10.3389/fpls.2020.00898

**Published:** 2020-06-16

**Authors:** Jun Liu, Xuewei Wang

**Affiliations:** Facility Horticulture Laboratory of Universities in Shandong, Weifang University of Science and Technology, Weifang, China

**Keywords:** deep learning, K-means, multiscale training, small object, object detection

## Abstract

Tomato is affected by various diseases and pests during its growth process. If the control is not timely, it will lead to yield reduction or even crop failure. How to control the diseases and pests effectively and help the vegetable farmers to improve the yield of tomato is very important, and the most important thing is to accurately identify the diseases and insect pests. Compared with the traditional pattern recognition method, the diseases and pests recognition method based on deep learning can directly input the original image. Instead of the tedious steps such as image preprocessing, feature extraction and feature classification in the traditional method, the end-to-end structure is adopted to simplify the recognition process and solve the problem that the feature extractor designed manually is difficult to obtain the feature expression closest to the natural attribute of the object. Based on the application of deep learning object detection, not only can save time and effort, but also can achieve real-time judgment, greatly reduce the huge loss caused by diseases and pests, which has important research value and significance. Based on the latest research results of detection theory based on deep learning object detection and the characteristics of tomato diseases and pests images, this study will build the dataset of tomato diseases and pests under the real natural environment, optimize the feature layer of Yolo V3 model by using image pyramid to achieve multi-scale feature detection, improve the detection accuracy and speed of Yolo V3 model, and detect the location and category of diseases and pests of tomato accurately and quickly. Through the above research, the key technology of tomato pest image recognition in natural environment is broken through, which provides reference for intelligent recognition and engineering application of plant diseases and pests detection.

## Introduction

The continuous development of economy and society has brought about global climate and environmental problems. The occurrence of diseases and insect pests seriously affects people’s life. The incidence and occurrence of plant diseases and insect pests is higher and higher and more complex (Food and Agriculture Organization of the United Nations,). Therefore, it is very important to study the prevention of plant diseases and insect pests, as well as the diagnosis and remedial measures of plant diseases and insect pests.

The origin of tomato is South America. Tomato is one of the important economic crops, which not only contains rich vitamins, but also can be used as fruit. In recent years, with the popularity of Western food, tomato sauce is more and more popular. The demand for tomato is increasing, and it has gradually become an important food in people’s daily life. Therefore, tomato plays an extremely important role in agricultural vegetable production and vegetable trade. As one of the most widely cultivated vegetables in the world, tomato has not only high yield, wide adaptability, but also high nutritional value. But, like other crops, tomato is affected by various diseases and pests in its growth process. Diseases include tomato virus disease, tomato nematode disease, tomato deficiency disease, tomato physiological disease, tomato bacterial disease, and tomato fungal disease. Pests mainly include leaf miner, greenhouse whitefly, Alfalfa noctuid moth, tobacco green worm, cotton bollworm, and *Polyphagotarsonemus latus*, etc. Usually, the occurrence of tomato diseases and pests is greatly affected by the local environment, varieties, cultivation, and management factors, so the types of tomato diseases and pests in different regions are different. China is one of the largest country in world vegetable production and export. The development of Shandong Shouguang tomato industry has a strong comparative advantage in the world. The cultivation area of facilities and open-field tomatoes has gradually increased. If improper field management can increase the probability of infection by diseases and pests, leading to outbreaks of diseases and pests, which has a significant impact on tomato yield and quality. Field investigation revealed that common tomato diseases in Shouguang, Shandong Province mainly include early blight, late blight, yellow leaf curl virus, brown spot, coal pollution, gray mold, leaf mold, navel rot, leaf curl disease, mosaic; common tomato pests mainly include leaf miner and greenhouse whitefly. According to the statistical data analysis of field investigation, it can be seen that the incidence of tomato diseases and pests in different farm households varies greatly in the field, with the yield loss of 1%–5% in the area with mild onset and more than 30% in the area with severe onset. At present, although many studies have reported the infection and detection of tomato pests and diseases in different regions, there is a lack of systematic reports on the detection of the main pests and diseases of tomatoes commonly found in Shouguang, Shandong Province. Therefore, it is very necessary to take 10 common diseases and 2 common insect pests in Shouguang area of Shandong Province as the research objects, and collect and collate the relevant data to provide theoretical basis for targeted early warning and prevention and control in the field.

The occurrence of tomato diseases and pests in different regions seriously affects tomato production. If the control is not timely, it will lead to yield reduction or even crop failure. Disease and pest prevention is the best way to reduce yield loss and reduce pesticide application to produce pollution-free vegetables. When plants grow to the point where symptoms of pests and diseases already occur, even if people can make accurate diagnosis and appropriate treatment, it is also a passive remedy. Although this is also very necessary, at this time, the application of agricultural chemicals and pesticides has poor control effect and is easy to cause environmental pollution, which leads to excessive pesticide residues in vegetables, and at the same time leads to more and more resistance of pests and diseases, making the work of crop disease resistance more and more difficult, which is an undesirable result. Therefore, early prediction and prevention of diseases and pests are very important. Research on tomatoes (Diaz-Pendon et al., 2010; Gilbertson and Batuman, 2013) shows how susceptible a plant is to be influenced by diseases and pests. With regard to how to effectively control diseases and insect pests and help vegetable farmers to improve the yield of tomato, the most important thing is to make accurate identification of diseases and insect pests. Therefore, the identification of tomato diseases and insect pests is the most serious challenge for scientific and technical personnel.

The traditional method of artificial detection of diseases and insect pests completely depends on the observation experience of the grower, or ask experts for guidance. Such a method is not only slow, but also is of low efficiency, high cost, strong subjectivity, low accuracy, and timeliness. With the continuous development of the Internet, the application of information technology provides new methods and ideas for crop diseases and insect pests’ identification. Using efficient image recognition technology can improve the efficiency of image recognition, reduce the cost, and improve the recognition accuracy. Therefore, experts and scholars at home and abroad have done a lot of research, in which deep learning has become the research focus. The application of deep learning in crop diseases and insect pests’ identification can greatly reduce the workload and shorten the identification time. Complex network structure and huge data samples are the biggest characteristics of deep learning. The emergence of deep learning technology provides strong technical support for image recognition.

Among them, the convolutional neural network (CNN) is a typical model of deep learning. The diseases and pests detection method based on CNN can automatically extract the features in the original image, which overcomes the subjectivity and limitation of artificial feature extraction in the traditional methods. The end-to-end structure simplifies the recognition process and solves the problems that manually designed feature extractor cannot get the feature expression closest to the natural attribute of the object. Based on the application of CNN object detection, not only can save time and effort, but also can achieve real-time judgment, greatly reduce the huge loss caused by diseases and pests, which has important research value and significance. Based on the latest research results of detection theory CNN object detection and the characteristics of tomato diseases and pests images, this study will build the dataset of tomato diseases and pests under the real natural environment, optimize the feature layer of Yolo V3 model by using image pyramid to achieve multi-scale feature detection, improve the detection accuracy and speed of Yolo V3 model, and detect the location and category of diseases and pests of tomato accurately and quickly. Through the above research, the key technology of tomato pest image recognition in natural environment is broken through, which provides reference for intelligent recognition and engineering application of plant diseases and pests detection.

In this paper, image data of tomato diseases and insect pests under real natural environment were collected, and corresponding data processing was carried out to build tomato diseases and pests detection dataset. Based on the YOLO v3 model as the main body, the image pyramid structure is adopted to fuse features of different levels to obtain feature maps of different scales for location and category prediction. Then, the dimension of the object box is clustered, and the number of anchor box is increased, so that the model can obtain more edge information of the object. Finally, in the training process, multi-size images are used for training, so that the model can adapt to images of different resolutions. Experiments show that the improved YOLO v3 algorithm can improve the detection speed while ensuring the detection accuracy.

## Related Work

### Comparison Between Traditional Machine Learning Technology and Deep Learning Technology

Before the development of deep learning technology, image recognition of plant diseases and insect pests was realized by traditional technology. With the development of deep learning technology, researchers begin to apply deep learning to the image recognition of diseases and pests, and have made a lot of achievements in recent years (Mohanty et al., 2016; Sladojevic et al., 2016; Wang et al., 2017; Liu et al., 2018; Ferentinos, 2018; Brahimi et al., 2018; Kaur et al., 2019), especially in the disease and insect image recognition of apple, tomato, cucumber, and other common crops. The efficiency and effect of image recognition are much better than that of traditional recognition methods. The comparison between traditional recognition method and deep learning recognition method is shown in **Table 1**.

**Table 1:** Contrast between traditional machine learning and deep learning.

Technology	Advantages	Disadvantages
Machine learning	It has advantages in training small data samples. No need of expensive hardware. The algorithm structure is simple and parameter adjustment is relatively simple.	Needs complex feature engineering and data dimensionality reduction. The classification accuracy is low and difficult for image recognition in complex background.
Deep learning	The deep feature extraction function makes it perform well in image, audio, and text data. Easy to update data through back propagation. Different architectures are suitable for different problems. The hidden layer reduces the dependence of algorithm on feature engineering.	The requirement of machine configuration is high. Needs massive data and relies on large-scale datasets.

### Object Detection Method of Plant Diseases and Insect Pests Based on CNN

Traditional machine vision methods have poor robustness in complex scenes, so it is difficult to meet the work requirements in complex scenes. The performance of CNN in image recognition has made great progress in the past few years, and in the ImageNet Large Scale Visual Recognition Challenge (ILSVRC), a lot of deep learning architectures have emerged, such as AlexNet (Krizhenvshky et al., 2012), GoogleLeNet (Szegedy et al., 2015), VGGNet (Simonyan and Zisserman, 2014), ResNet (Xie et al., 2017), and the accuracy of general image recognition is constantly being refreshed. CNN’s hard work and great breakthrough in the large-scale image classification competition prompted people to consider applying it to the problem of object detection. Different from image classification, object detection needs to detect and locate specific multiple objects from the image, which is mainly divided into two categories. One is to generate a series of candidate frames as samples by the algorithm, and then classify the samples by CNN, such as RCNN (Girshick et al., 2014), faster RCNN (Ren et al., 2016), and R-FCN (Dai et al., 2016). The other one directly transforms the problem of object bounding box location into regression problem, which does not need to generate candidate boxes. The landmark algorithms include SSD (Liu et al., 2016) and YOLO (Redmon et al., 2016).

Arsenovic et al. (2019) established a plant disease dataset with 79,265 pieces collected under different meteorological conditions. A two-level structure, PlantDiseasenet, which is trained by PDNet-1 and PDNet-2 at the same time, is proposed. PDNet-1 uses the detection method proposed in the Yolo algorithm as the detection tool of plant leaves, and PDNet-2 is responsible for the classification of plant leaves. After training, the accuracy of the model is 93.67%. Jiang et al. (2019) proposed an improved CNN-based deep learning method for real-time detection of apple leaf diseases and insect pests. Firstly, through data expansion and image annotation technology, an apple leaf disease dataset (ALDD) composed of laboratory images and complex images under real field conditions is constructed. On this basis, a new method is proposed by introducing GoogleNet inception structure and rainbow concatenation. Finally, the proposed INAR-SSD (SSD with perception module and rainbow condition) model was trained to detect the five common apple leaf diseases and insect pests. The experimental results show that the model achieved 78.80% mAP and the detection speed is as high as 23.13 FPS. Tian et al. (2019) proposed a method of Apple Anthracnose damage detection based on deep learning and optimized the feature layer of Yolo V3 model with low resolution by using Densenet, which has greatly improved the utilization of neural network features and improved the detection results of Yolo V3 model. The experimental results show that the model achieved 95.57% mAP. Zheng et al. (2019) established a CropDeep species classification and detection dataset, including 31,147 images and more than 49,000 annotation examples from 31 different categories. These images are collected in real natural environment with different cameras, and the most advanced deep learning classification and detection model is used to provide a wide range of baseline experiments. The results show that the existing classification method based on deep learning can achieve more than 99% in classification accuracy, and only 92% in object detection accuracy. Meanwhile, Yolo V3 model has good application potential in agricultural detection tasks.

### Object Detection of Tomato Diseases and Insect Pests Based on CNN

Fuentes et al. (2017) proposed a real-time system for the identification and location of tomato plant diseases and insect pests. The system is consisted of three network structures, Fast R-CNN, SSD, and R-FCN are compared. Each network structure is combined with different feature extraction networks (such as VGG, RESNET). The system has the following advantages: (1) the images used in the system are collected in the field; (2) considering that a crop may be affected by multiple diseases and insect pests at the same time; (3) the system has strong robustness and can be used in the actual field environment to achieve detection; (4) the system can also solve complex tasks, such as infection status (early, late), infection location (leaves, culm, fruit) and both sides of leaves (obverse and reverse). Fuentes et al. (2018) proposed an improved framework of tomato plant diseases and insect pests detection algorithm aiming at the problem of false alarm and unbalanced classification, which is composed of three main units: (1) the main diagnosis unit (bounding box generator), which generates the bounding box containing the location of infected area and class; (2) the auxiliary diagnosis unit (CNN filter bank), which trains each independent CNN to filter the samples of misclassification; (3) the integration unit, which combines the information of the autonomous diagnosis unit and the auxiliary diagnosis unit, and keeps the true positive samples at the same time, so as to eliminate the false positive of the classification errors in the first unit. The experiment shows that the recognition rate of this method is about 96%. Fuentes et al. (2019) proposed a method which can not only effectively detect and locate plant anomalies, but also generate diagnostic results, display the location of anomalies and describe the symptoms of sentences as output, and has achieved 92.5% average precision (mAP) in the newly created tomato plant anomaly description dataset. However, it uses Faster R-CNN to detect the object, which needs to be carried out in two steps. First, extract the region proposal, and then detect it. However, Yolo directly generates coordinates and probability of each category through expression once. Therefore, the real-time performance of existing research needs to be improved.

### Problems of Existing Research and Development Trends

The development of the existing research provides a reference and feasibility basis for the application of CNN in the detection of plant diseases and pests, and can avoid the shortcomings of the traditional machine vision method in the feature extraction process. Through CNN object detection technology, it can greatly facilitate plant breeding and help farmers to supervise their fields better. However, the automatic identification and detection of plant diseases and insect pests are mainly faced with the following difficulties (Barbedo, 2016a; Barbedo, 2016b; Barbedo, 2017; Barbedo, 2018a; Barbedo, 2018b).

- The background of the image is complex. In addition to the infected leaves, the image may contain other elements, such as crop stalks, soil, etc. Especially for the photos taken in the field environment, not only the background is messy, there are also factors such as light, angle difference, etc.
- There may be no clear boundary between the infected area and the healthy area.
- The same disease has different characteristics in different stages of development, even in different locations.
- The characteristics of different kinds of diseases and insect pests may be the same or slightly different, and there may be multiple diseases and insect pests at the same location at the same time.
- It is difficult to distinguish the infected area from other dead plant tissues.
- Throughout the different stages of development of the insect (nymph or larva and adult) and the different instars, the morphological characteristics of insect vary greatly. Especially for small target pests, it is difficult to identify.

At present, the research of plant diseases and insect pests based on deep learning involves a wide range of crops, including all kinds of vegetables, fruits, and food crops. The completed tasks not only include the basic tasks of classification and detection of diseases and insect pests, but also more complex tasks such as the judgment of infection degree. Because deep learning relies on large-scale datasets, there are few open datasets of plant diseases and insect pests at present, the researchers usually find the best solution by comparing different training set and test set proportion and using different network models. However, there is still a certain gap between the complexity of these susceptible images and the real field scene, and there is a certain gap between the real-time detection of diseases and pests in fields based on mobile devices.

## The Principle Of Yolo V3 Model

### Yolo V3 Design Idea

Yolo algorithm proposed by Redmon et al. (2016) in 2016, the object detection task is transformed into regression problem, which greatly speeds up the detection speed. Yolo V3 (Redmon and Farhadi, 2018) was proposed on the basis of Yolo V2 (Redmon and Farhadi, 2016), the detection speed of Yolo V2 is maintained, and the detection accuracy is greatly improved. Yolo V3 uses the idea of residual neural network (He K et al., 2016). The introduction of multiple residual network modules and the use of multi-scale prediction improve the shortcomings of Yolo V2 network in small object recognition. Because of the high accuracy and timeliness of detection, this algorithm is one of the best algorithms in the field of object detection. This model uses a lot of 3 × 3 and 1 × 1 convolution layers with good performance, and some residual network structures are also used in the subsequent multi-scale prediction. Finally, it has 53 convolution layers, so it can also be called Darknet-53.

Yolo V3 network introduces the idea of using anchor boxes in Faster R-CNN. For coco dataset and VOC dataset, three scales are used for prediction. Each scale has three anchor boxes, and the feature map with large scale uses small priori box, so you can select the appropriate priori box according to the target you want to identify, and the network structure can be changed according to the scale that needs to predict.

### Feature Extraction Network Darknet-53

**Figure 1:**
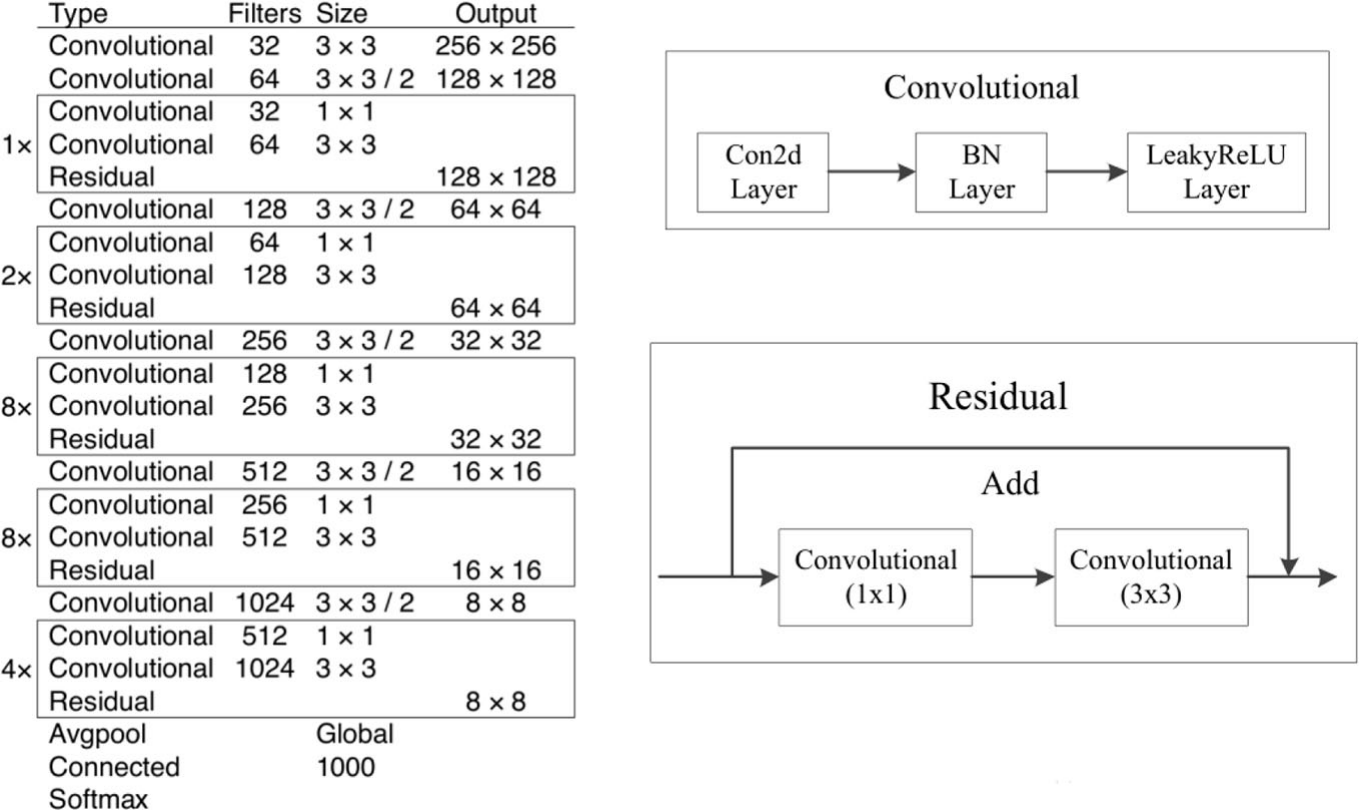
Darknet-53 network structure.

Yolo V3 adopts the network structure of Darknet-53, which consists of 53 (2 + 1*2 + 1 + 2*2 + 1 + 8*2 + 1 + 8*2 + 1 + 4*2 + 1 = 53) convolution layers. The network structure contains 53 convolutional layers and 5 maximum pooling layers. Batch normalization and dropout operations are added after each convolutional layer to prevent overfitting. Darknet-53 is composed of five residual blocks, which uses the idea of residual neural network for reference. **Figure 1** shows the Darknet-53 network structure. Yolo V3 has increased the network depth by introducing residual unit to avoid gradient disappearance.

## The Method Of Improving Yolo V3 Model

**Figure 2:**
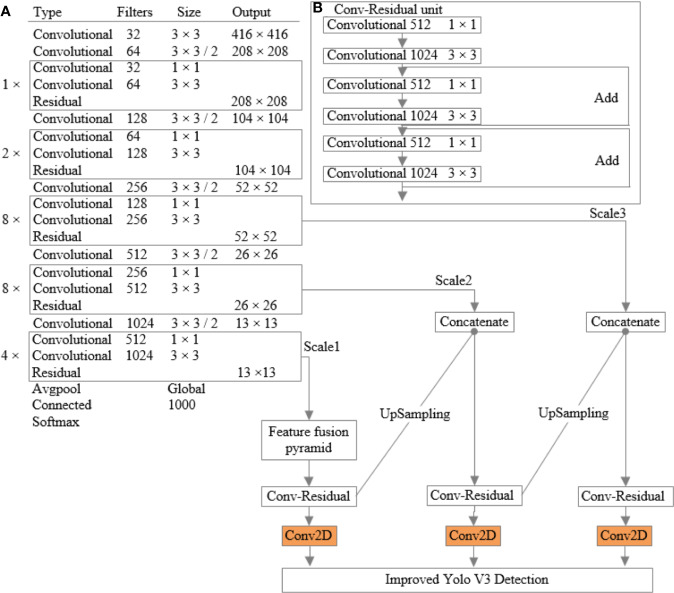
The improved Yolo V3 network structure **(A)** network structure; **(B)** conv-residual unit.

Compared with the whole tomato plant image, the disease spots and pests are small objects. Therefore, Yolo V3 model is a little inadequate in the scale when recognizing tomato disease spots and pests. In view of this situation, Yolo V3 model is improved to adapt to specific tomato diseases and pests detection tasks. The improved Yolo V3 network structure is shown in **Figure 2**.

The improved Yolo V3 network structure uses feature fusion to increase the number of feature maps, which saves a lot of computation and is very important for improving the running speed. Two residual units were added to the detection network to further enhance the expression of image features. Also, the image pyramid structure and improved conv-residual unit are used to reduce the impact of depth reduction on the precision of the backbone network. Therefore, the improved Yolo V3 network structure inherits Darknet-53’s structure in ideology but reduces the dependence of the whole network on ResNet, and optimize the network forward propagation speed and model size, making it more suitable for mobile platforms with weak computing power.

Based on the image pyramid structure, Yolo V3 model is improved. Upper sampling is used to fuse the high-level features with the low-level features, and three sets of prediction feature maps of different scales are obtained. Then, the location and category are predicted on these three sets of prediction feature maps.

K-means algorithm is used to calculate the prior box dimension in the self-built tomato diseases and pests dataset. The priori box dimension obtained in Yolo V3 model is trained on the COCO dataset, and its parameters are divided into three different scales. For the specific task of tomato diseases and pests detection, cluster operation should be carried out for the specific dataset, and smaller priori box should be used for the larger scale feature map to get the corresponding cluster center.

The multi-scale training strategy was used to train the self-built dataset of tomato diseases and insect pests. In Yolo V3 model, the full connection layer is removed and uses the full convolution operation. In the training process, the input size can be changed at any time, so that the trained model can adapt to different scales of tomato diseases and pests images.

### Multiscale Feature Detection Based on Image Pyramid

In Yolo V3, Darknet-53 network is used to extract features, and feature visualization technology is used to clearly show the effect of each level of features. Low-level features have rich details and location information, while high-level features have rich semantic features. From low-level to high-level, the details are decreasing, while the semantic information is increasing. For location prediction, more low-level feature information is needed, for category prediction, more high-level local information is needed. Therefore, based on the image pyramid model, the up sampling method is used to fuse the high-level features with the low-level features to obtain the feature maps of different scales for location and category prediction.

**Figure 3:**
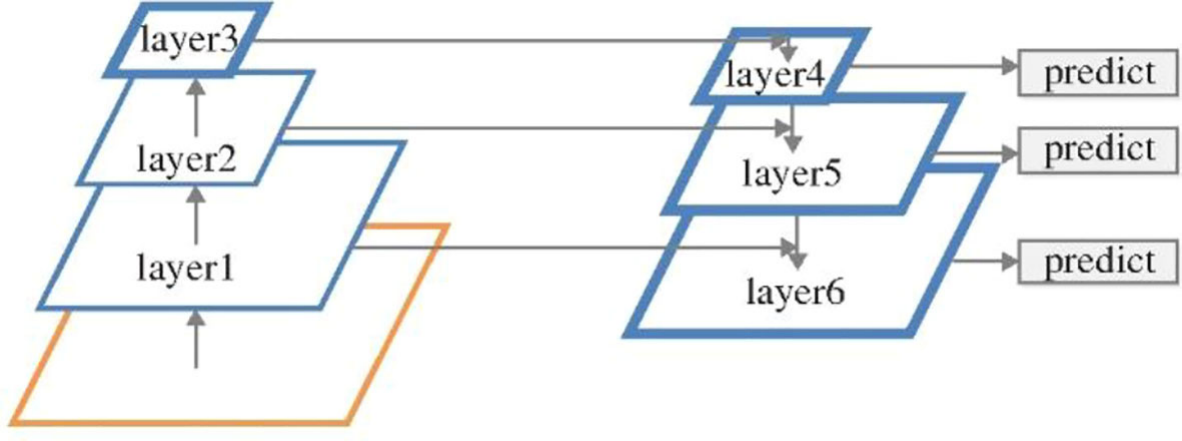
Feature Fusion Pyramid.

The feature pyramid on the right side of **Figure 3** is generated by the feature pyramid on the left side. The whole process is as follows: first, the input image is deeply convoluted, then the features on layer2 are convoluted, and the features on layer4 are sampled to make them have the same size. Then, the processed layers layer2 and layer4 are convoluted and operated to input the obtained results to layer5. In the same way, feature fusion is carried out among multiple layers to get multiple sets of feature maps for prediction. Based on this scheme, the processed low-level features and high-level features are accumulated. The purpose of doing this is that because the low-level features can provide more accurate location information, and multiple down sampling and up sampling operations make the location information of the deep-seated network have errors. Therefore, they are combined to build the deep feature pyramid, and integrate the multi-layer feature information on different feature maps for category and location prediction.

**Figure 4:**
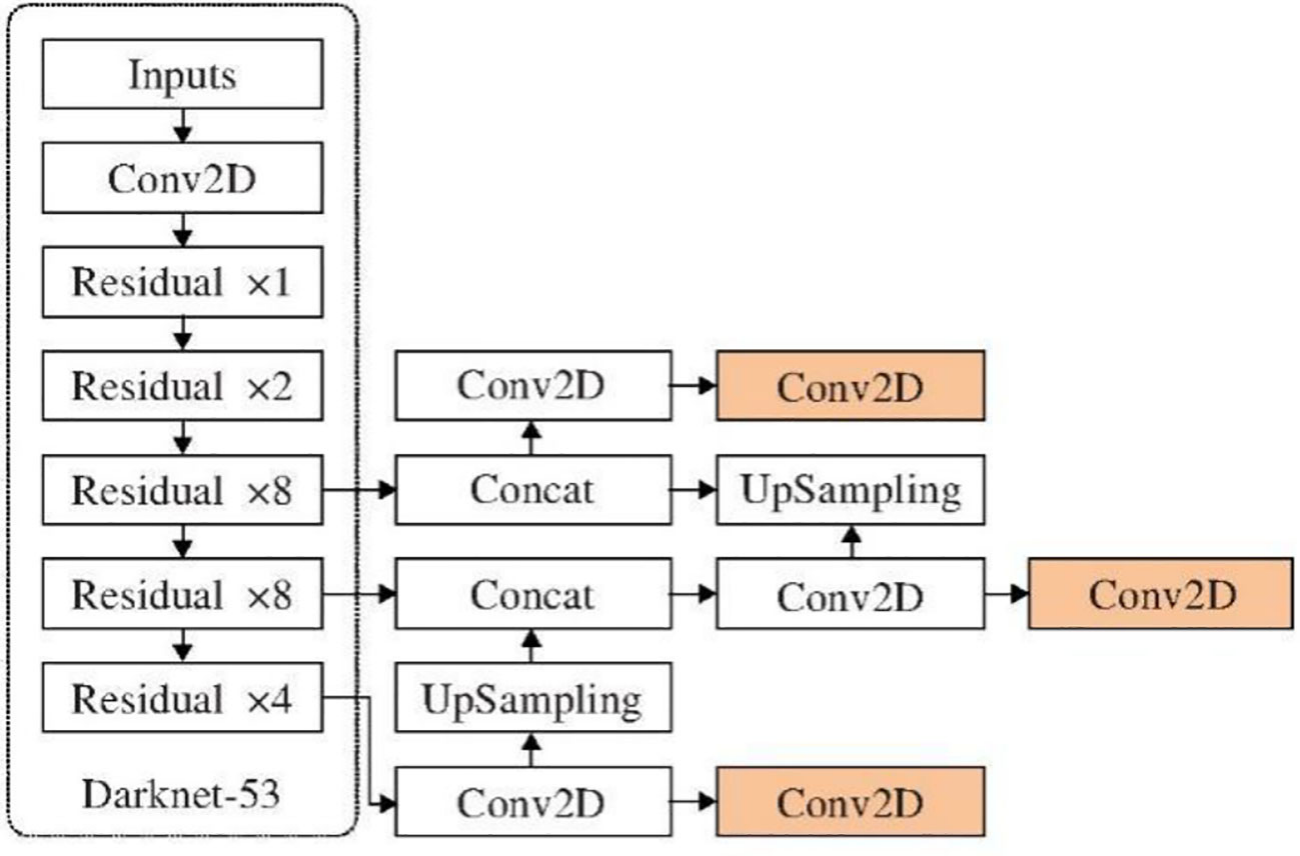
Improved network structure.

Based on the above idea of feature fusion, Yolo V3 algorithm is improved. Up sampling is used to fuse high-level features with low-level features. Finally, three sets of feature maps are obtained, and these three sets of feature maps of different scales are used for prediction. The improved network structure is shown in **Figure 4**.

Specific network structure improvement details: first, get the characteristic pyramid through the Darknet-53, and carry out continuous 1 × 1 and 3 × 3 convolution operations on the conv53 layer to get a group of Yolo layers to be processed, and then carry out a group of 1 × 1 and 3 × 3 convolution operations on the layer to get a small scale Yolo layer. At the same time, perform upper sampling operation on this layer, and carry out convolution operation with the conv45 layer in the Darknet-53, as well using continuous 1 × 1 and 3 × 3 convolution operations, the second group of Yolo layers to be processed are obtained, and a group of 1 × 1 and 3 × 3 convolution operations are performed on this layer to obtain the mesoscale Yolo layer. At the same time, perform upper sampling operation on this layer, convoluted and operated with conv29 layer in Darknet-53, and the third group of Yolo layers to be processed are also obtained by using continuous 1 × 1 and 3 × 3 convolution operations, and a group of 1 × 1 and 3 × 1 layers are performed on this layer 3 convolution operation to get large scale Yolo layer. After the above operations, three sets of Yolo feature layers with different scales are obtained, and these three sets of feature layers are used for location and category prediction.

### K-Means Dimension Clustering Algorithm

Yolo V3 uses the prior box to predict the coordinates of the bounding box in Yolo V2. The difference is that Yolo V3 uses k-means algorithm to predict and gets nine prior boxes, and divides them into three scale feature maps, among which the larger scale feature map uses the smaller prior box to obtain more edge information of the object.

The dimensions of nine sets of prior boxes calculated in Yolo V3 are respectively as follows: (10, 13), (16, 30), (33, 23), (30, 61), (62, 45), (59, 119), (116, 90), (156, 198), and (373, 326). However, in the actual detection task of tomato diseases and insect pests, the prior box dimension calculated by Yolo V3 algorithm is not suitable for the detection scene of tomato diseases and insect pests, so it is difficult to get accurate object bounding box information by using the original prior box dimension in Yolo V3 algorithm.

Therefore, in the tomato diseases and pests detection scenario, K-means algorithm is used to cluster the self-built tomato diseases and pests dataset, and nine sets of prior box dimension centers are obtained respectively as follows: (44, 11), (53, 18), (60, 22), (73, 25), (78, 27), (81, 31), (89, 41), (97, 43), and (109,51). The region is arranged from small to large, and divided into feature maps of three different scales, among which the larger scale feature map uses the smaller prior frame. Finally, the cluster center is used to detect tomato diseases and insect pests.

### Multiscale Training

In the Yolo detection algorithm, convolutional network is used to extract the features, and then the full connection layer is used to get the prediction value. However, due to the existence of full connection layer, the input image size of the network must be fixed in the training process, so the final training network does not have robustness to different sizes of test images.

**Figure 5:**
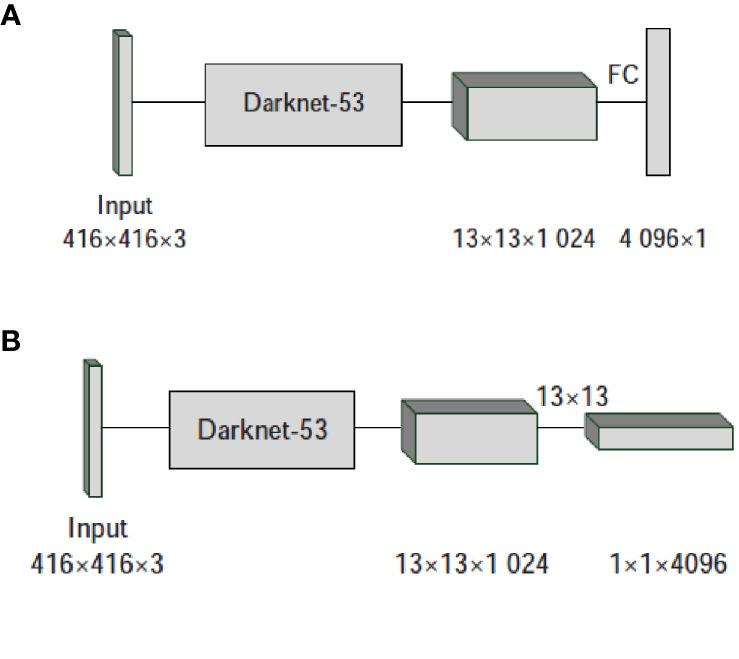
Conversion of full connection layer to convolution layer. **(A)** Full connection operation. **(B)** Convulation operation.

According to the self-built tomato diseases and pests dataset, the size of the input image is different. Therefore, in order to enhance the robustness of the model to different image sizes, multi-scale training strategy is adopted. Specifically, remove the full connection layer in Yolo V3 network and change it to full convolution operation. **Figure 5** shows the process of converting a full connection layer to a convolution layer.

**Figure 5A** uses the full connection layer for prediction, and **Figure 5B** converts the full connection layer into a convolution layer for prediction. When the input image size is 416 × 416, after passing through the Darknet-53 network, 13 × 13 × 1024 feature maps are output. Passing through a full connection layer containing 4,096 neurons, a set of 4,096 × 1 feature maps are obtained in **Figure 5A**. **Figure 5B** uses 4,096 convolution kernels of 13 × 13, and finally obtains 1 × 1 × 4,096 feature maps, which is essentially equivalent to 4,096 nerves obtained by full connection. For the above two network structures, when the image input size is 416 × 416, the network can operate normally, but there are other sizes of pictures in the self-built tomato diseases and pests dataset. For example, when inputting 608 × 608 size images, after passing through the Darknet-53 network, the 19 × 19 × 1024 feature map is output. For the structure of **Figure 5A**, the next step is to connect the feature map with 4,096 neurons. The size of the original architecture is 13 × 13, and now it is 19 × 19. Therefore, during the propagation of the network, the previous parameter matrix cannot be used, and the network cannot operate normally. For the structure of **Figure 5B**, after the full connection is changed to convolution operation, the network can continue to run, and finally get the correct output of 7 × 7 × 4,096. Therefore, after the full connection layer is changed to full convolution operation, input images of different sizes are used for training, and the improved algorithm can adapt to different sizes of test images.

In addition, after changing the full connection layer to full convolution operation, the performance of the network will be improved. When using the full connection layer in **Figure 5A** in the forward propagation operation of the network, it can be calculated that 708,837,377 parameters are needed. While using the full convolution operation in **Figure 5B**, only 696,320 parameters are needed. Therefore, after converting the full connection layer into convolution operation, the number of parameters can be reduced, the network operation amount can be reduced, and the network performance can be improved.

Based on the above analysis, this paper will use multi-scale training strategy to train the self-built tomato diseases and pests dataset. Because the whole network has five maximum pool layers, the down sampling rate of the network is 32. In the training process, the input size of the training picture of the dataset of tomato diseases and pests is divided into a series of values of multiple of 32, and the calculation formula of the size is as follows:

$$S_{n+1}=32+S_{n’}n\leq 9\left(1\right)$$

Among them, *S_n_* is the size of the group *n* input image. During network initialization, *S*_1_ is 320 × 320. Through formula (1), it can be concluded that the size of the input image is: 320, 352, 384, 416, 448, 480, 512, 544, 576, and 608. In the process of training, one kind of input image size is selected randomly every 10 rounds to achieve the effect that the model can adapt to images of different sizes.

## Experimental Results And Comparative Analysis

### Experimental Operation Environment

The experimental hardware environment of this paper is shown in **Table 2**. On this basis, the software environment is built as follows: Ubuntu 16.04, python, OPPENCV, CUDA, etc. The framework uses the Caffe framework and Darknet-53 framework.

**Table 2:** Configuration of experimental hardware environment.

Hardware name	Model	Number
Main board	Asus WS X299 SAGE	1
CPU	INTEL I7-9800X	1
Memory	The Kingston 16G DDR4	2
Graphics card	GEFORCE GTX1080Ti	2
Solid state drives	Kingston 256G	1
Hard disk	Western digital 1T	1

### Experimental Dataset Building

In the process of building tomato diseases and pests detection dataset, different equipment including monitoring, digital camera, and smart phone are used to collect tomato plant photos in the local tomato planting greenhouses. In different time periods, different weather conditions and different scenes, single frame images and photos in the monitoring video are randomly selected as the dataset. The number of samples of each category is shown in **Table 3**. The labeling tool is used to mark the images. With this tool, when the manual operation is carried out, we only need to mark the user-defined objects in the image, and the tool can automatically generate the corresponding configuration file.

**Table 3:** Number of samples of each disease type.

Serial Number	Disease/Pest	Sample size	Number of labeled samples (bounding box)	Percent of bounding box samples
1	Early blight	1,209	12,187	8.30%
2	Late blight	1,303	12,362	8.41%
3	Yellow leaf curl virus	1,286	12,138	8.26%
4	Brown spot	1,348	11,726	7.98%
5	Coal pollution	1,287	13,025	8.87%
6	Gray mold	1,263	12,184	8.29%
7	Leaf mold	1,377	12,399	8.44%
8	Navel rot	1,106	12,026	8.19%
9	Leaf curl disease	1,198	11,734	7.99%
10	Mosaic	1,242	13,092	8.91%
11	Leaf miner	1,228	11,580	7.88%
12	Greenhouse whitefly	1,153	12,459	8.48%
	Total	15,000	146,912	100.00%

### Model Training

Use the weight parameters provided on Yolo V3 official website as the initialization parameters of network training, randomly use the images in the self-built tomato diseases and pests detection training dataset to finetune the network parameters, so that the detection effect of the whole model is optimal, and the model parameter settings are shown in **Table 4**.

**Table 4:** Model parameter settings.

Name	Value
Batch Size	64
Learning Rate	0.01
Epoch	13,000
Momentum	0.9
Match Threshold	0.5
NMS	0.3

### Comparative Analysis of Experiments

In this paper, Yolo V3 algorithm is mainly used for the experiment. The improvements include multi-scale feature detection based on image pyramid, object frame dimension clustering, and multi-scale training. At the same time, in order to verify the validity, accuracy and stability of the model, SSD, Faster R-CNN, and Yolo V3 algorithms are used for experimental comparative analysis, and the detection accuracy mAP (Mean Average Precision) and detection time are used as the evaluation indexes of detection effect. The results are shown in **Table 5**.

**Table 5:** Comparison of experimental results.

Algorithm name	Accuracy (%)	Time/(ms)
SSD	84.32	25.69
Faster R-CNN	90.67	2868.94
Yolo V3	88.31	21.18
Improved Yolo V3	92.39	20.39

It can be seen from the experimental results that in terms of object detection accuracy, Faster R-CNN and the improved Yolo V3 are superior to the other two algorithms. However, due to the need to establish RPN network in the process of object detection, which involves a lot of calculation, Faster R-CNN is inferior to the improved Yolo V3 in detection speed. In general, the improved Yolo V3 algorithm is superior to the other three algorithms in detection accuracy and speed, especially the detection time is the shortest. Therefore, the improved Yolo V3 performs best for real-time detection tasks. The improved Yolo V3 algorithm can complete the detection task of tomato diseases and insect pests well on the premise of considering both the detection accuracy and detection speed.

**Figure 6:**
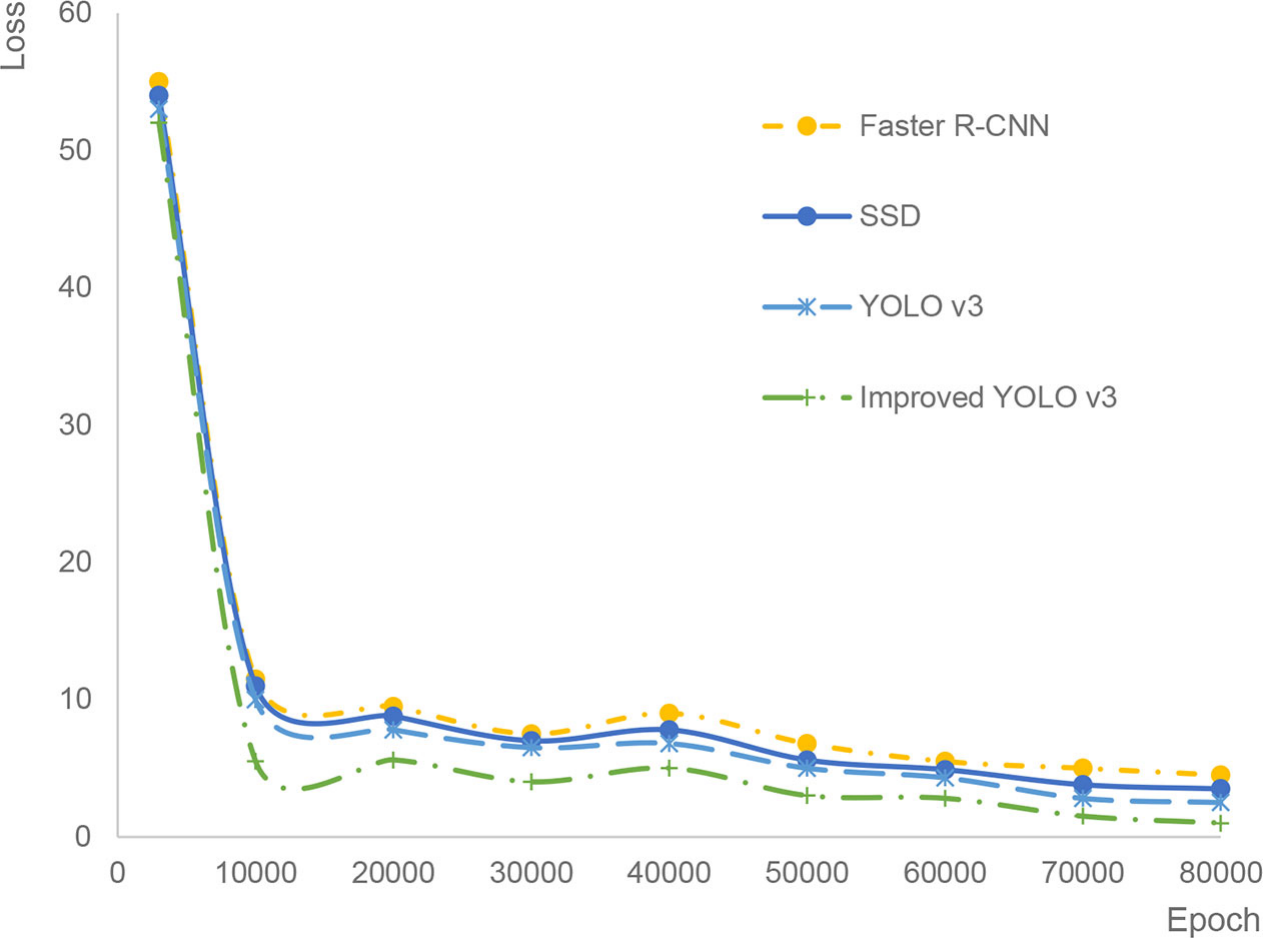
Loss function contrast.

The loss curves of the four algorithms are shown in **Figure 6**. It can be seen that the loss of the algorithm in this paper is the smallest, while Faster R-CNN is the largest. Therefore, the convergence speed of this algorithm is the fastest.

### Performance Analysis in Small Object Scenario

**Figure 7:**
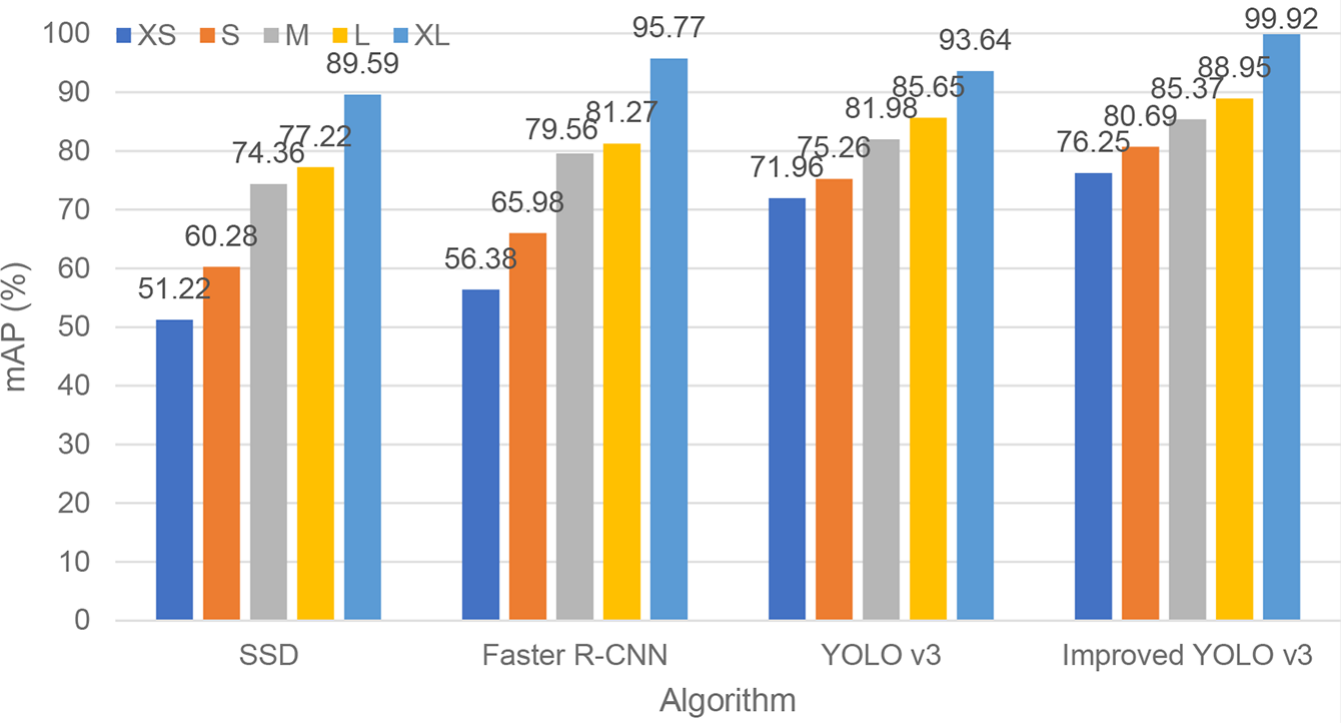
Object size sensitivity analysis of four algorithms.

Performance analysis in small object scenario is important for different sizes of diseases and pests objects throughout their different stages. The multi-scale detection method based on image pyramid proposed in this paper improves the detection effect of small objects. In order to verify the detection effect of the small object detection performance, the test dataset is sorted according to the size of the object. The 0-10%, 10% - 30%, 30% - 70%, 70% - 90% of the object size are divided into five sub categories: XS, S, M, L, XL, which represent the size of different objects. **Figure 7** shows the detection performance of the original Yolo V3 algorithm and the improved algorithm for objects of different sizes.

As can be seen from **Figure 7**, the detection accuracy of the improved Yolo V3 algorithm for objects of different sizes is higher than that of the other three algorithms. Therefore, when detecting tomato diseases and insect pests, using the strategy of multiscale feature fusion, combining the high-level features with the low-level features, the improved algorithm has achieved the best results in the detection of small objects. Throughout the different stages of development of the insect (nymph or larva and adult) and the different instars, the morphological characteristics of insect vary greatly. For small object pests, the improved algorithm can achieve good results.

### Performance Analysis of Different Resolution Images

**Figure 8:**
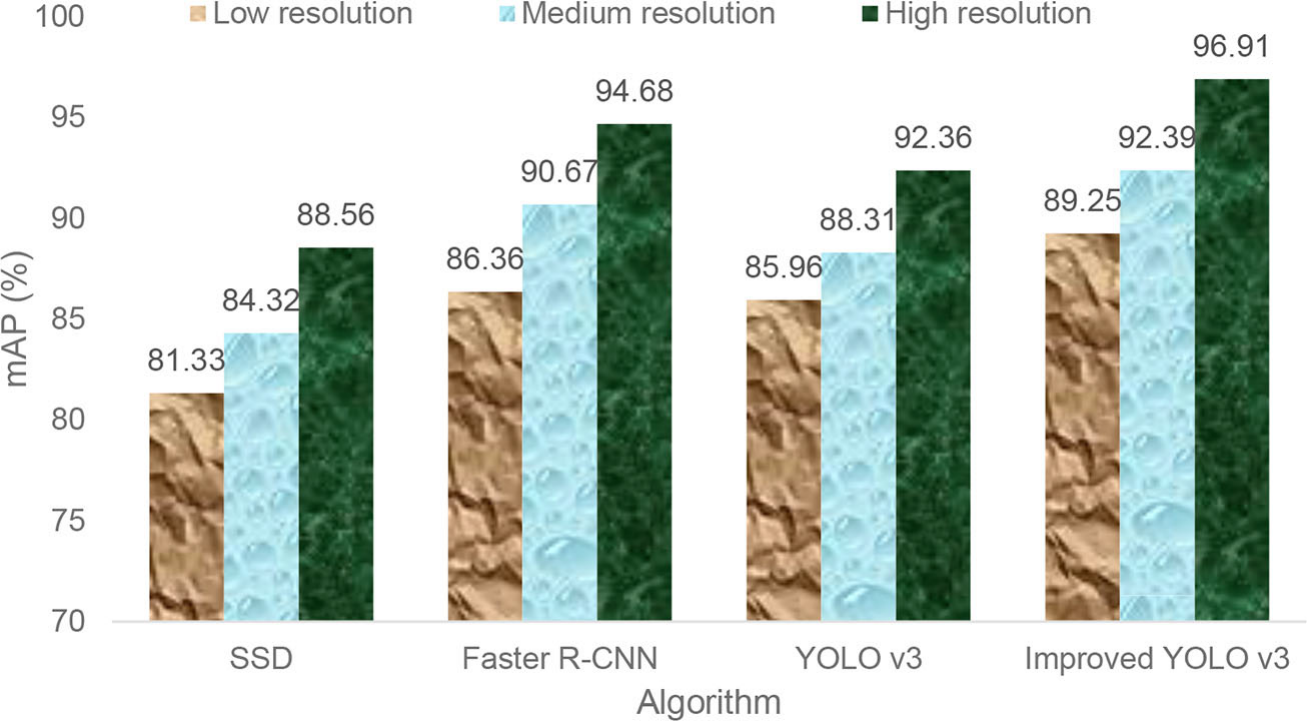
Object image resolution sensitivity analysis of four algorithms.

The multi-scale training method proposed in this paper can enhance the robustness of the model for the detection of different resolution images. In order to verify the detection effect of the model on the input images of different resolutions, this paper divides the test dataset images into three different resolution sizes, namely {320, 608, 1024}, representing three types of images: low resolution, medium resolution and high resolution. **Figure 8** represents the detection accuracy performance of four algorithms for different resolution images.

As can be seen from **Figure 8**, the detection accuracy of the improved Yolo V3 algorithm for different resolution images is higher than that of the other three algorithms. It can be seen that the multi-scale training detection strategy adopted in this paper not only can enhance the adaptability of the model to different resolution images, but also can detect the location and category of tomato diseases and insect pests accurately and quickly.

**Figure 9:**
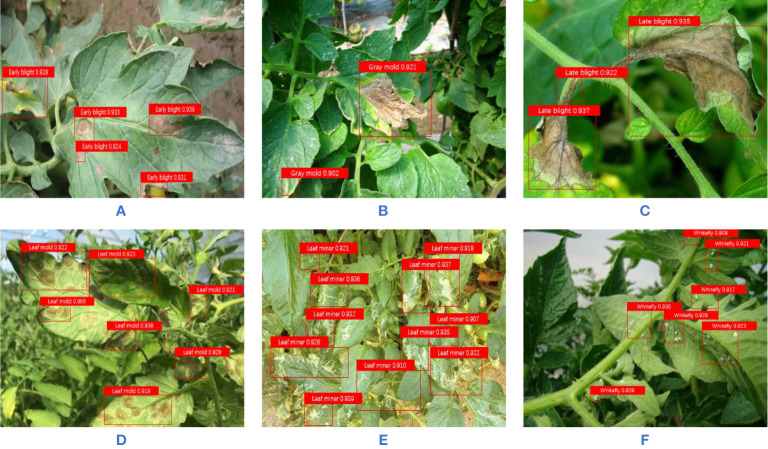
The detection effect diagram of the improved YOLO v3 algorithm **(A)** Early bight; **(B)** Gray mold; **(C)** Late blight; **(D)** Leaf mold; **(E)** Leaf miner; **(F)** Whitefly.

In addition, in order to show the detection effect of the algorithm in this paper more intuitively, some detection images are selected, as shown in **Figure 9**. It can be seen that the algorithm in this paper can correctly detect the location and category of the object when there are multiple objects and small objects in the image, and can effectively avoid the problem of false detection and missing detection.

## Conclusions and Future Directions

### Conclusions

1. In this paper, an improved Yolo V3 algorithm is proposed to detect tomato diseases and insect pests. Yolo V3 network was improved by using multi-scale feature detection based on image pyramid, object bounding box dimension clustering and multi-scale training. The experimental results show that the detection accuracy of the algorithm is 92.39% and the detection time is only 20.39 ms. Therefore, for the task of tomato diseases and pests detection, the improved Yolo V3 algorithm proposed in this paper can not only maintain a high detection rate, but also meet the real-time detection requirements, and can detect the location and category of tomato diseases and insect pests accurately and quickly.
2. Compared with SSD, Faster R-CNN and the original Yolo V3, the improved Yolo V3 CNN can achieve higher detection accuracy and shorter detection time, and meet the requirements of real-time detection accuracy and speed of tomato diseases and pests.
3. Performance analysis in small object scenario and different resolution images further verifies that the improved Yolo V3 network has strong robustness for detection of different object sizes and different resolution images in complex environment, and has high detection and positioning accuracy, which can meet the needs of tomato diseases and pests detection in complex environment.

### Future Directions

1. In different growth cycles, the appearances of diseases and pests are different. Therefore, the images of diseases and pests should be divided more carefully, and the same class of diseases and pests should be divided according to the growth period as the standard. In the future, the division of dataset will be improved.
2. In order to make the model more widely applied, the next work will collect a large number of high-quality images of different types of diseases and pests, and proceed to insert other insect pests, optimize and adjust the model, and extend this to other crops, so as to improve the practicability and accuracy of crop diseases and pests image recognition.
3. Construct an intelligent patrol robot for tomato diseases. In a real greenhouse tomato planting base, the intelligent patrol inspection robot for Tomato Diseases can work around the clock. The utility of the improved Yolo V3 model will be further enhanced by carrying a mobile robot arm with a detection sensor for disease detection. The purpose of early detection and early diagnosis of tomato diseases will be achieved by capturing the lesions in real time and non-destructive detection of tomato diseases.

## Data Availability Statement

The datasets analyzed in this article are not publicly available. Requests to access the datasets should be directed to liu_jun860116@wfust.edu.cn.

## Author Contributions

JL designed research. JL and XW conducted experiments and data analysis and wrote the manuscript. XW revised the manuscript. All authors contributed to the article and approved the submitted version.

## Funding

This study is supported by the Facility Horticulture Laboratory of Universities in Shandong with project numbers 2019YY003, 2018RC002, 2018YY016, 2018YY043, and 2018YY044; Research and Development Plan of Applied Technology in Shouguang with project number 2018JH12; 2018 innovation fund of science and technology development center of the China ministry of education with project number 2018A02013; 2019 basic capacity construction project of private colleges and universities in Shandong province; Weifang Science and Technology Development Program with project numbers 2019GX081 and 2019GX082. Key research and development plan of Shandong Province with project number 2019RKA07012 and 2019GNC106034.

## Conflict of Interest

The authors declare that the research was conducted in the absence of any commercial or financial relationships that could be construed as a potential conflict of interest.
